# The association between different family environments and adolescent depressive symptoms: the mediating effects of internet addiction and peer relationships

**DOI:** 10.3389/fpsyt.2025.1719348

**Published:** 2025-11-24

**Authors:** Haiqing Feng, Kefan Jiang, Yufei Lu, Dingmeng Liu, Aihui Zhu, Guangzhong Zhou, Yuexia Gao, Qingyun Lu

**Affiliations:** 1 Department of Health Management, School of Public Health, Nantong University, Nantong, Jiangsu, China; 2 Xiu Fu Junior High School, Yancheng, Jiangsu, China

**Keywords:** Family emotional environment, Family economic environment, adolescents, depressive symptoms, Internet addiction, peer relationships

## Abstract

**Introduction:**

This study aims to examine the relationship between different family environments and depressive symptoms in adolescents, with a particular focus on the association between the family emotional environment and depressive symptoms, as well as the mediating effects of internet addiction and peer relationships.

**Methods:**

Using cluster sampling, 2,825 adolescents were surveyed using the questionnaire on basic family circumstances, the parenting style scale (PBI), the children’s perception of Interparental conflict scale (CPIC), the Internet addiction diagnosis scale (YDQ), the peer relationship scale, and the child depression scale (CDI).

**Results:**

(1) The family emotional environment can be divided into low emotional support families (52.4%) and high emotional support families (47.6%), and the family economic environment can be divided into low socioeconomic status (SES) families (71.3%) and high SES families (28.7%);(2) The family emotional environment was linked to depressive symptoms in adolescents (r= - 0.07, *P* < 0.01), while the family economic environment was not (*P*>0.05). (3) Internet addiction and peer relationships mediated the relationship between family emotional environment and adolescent depressive symptoms after controlling for gender, home location, and only-child status.

**Discussion:**

The study results emphasize the importance of creating a positive emotional environment at home, which is an effective measure for alleviating symptoms of depression in adolescents, reducing the risk of internet addiction, and improving the quality of peer relationships.

## 1 Introduction

Depression ranks as the fourth leading cause of global adolescent disability and the second leading cause of death (1). In 2020, the detection rate of depressive symptoms among Chinese adolescents was 24.6%, with a 7.4% detection rate for serious depression (2). Family, as the core environment for individual growth and development, is closely related to adolescents’ depressive symptoms (3). Generally speaking, a positive family environment can reduce the effects of stress and promote mental health (4). However, previous studies on the family environment have certain limitations. They usually discuss the family environment as a whole in general terms or focus only on a specific aspect. This type of singular or isolated research perspective has obvious shortcomings. The factors it focuses on either have a low prevalence in the general population or can only cover a small part of the family environment. In fact, the concept of family environment is broad. According to existing research, it can be clearly divided into the family emotional environment and family economic environment (5). Therefore, this study adopts a multidimensional conceptualization of the family environment, combining multiple sub scales, with the aim of exploring the mechanisms through which different aspects of the family environment influence individuals and providing more targeted recommendations and strategies for future family interventions. However, how to best characterize the cognitive specificity and influence mechanisms of the family emotional and economic environment across different subgroups of depression remains a controversial issue. Using appropriate statistical techniques to draw conclusions on this question is crucial. In light of the research objectives and data characteristics, this study ultimately employs a two-step cluster analysis. The rationale is as follows: Firstly, the core purpose of this study is to identify the intrinsic sub types of family socioeconomic status, rather than obtaining a continuous composite score to verify a theoretical structure. As an exploratory data-driven technique, two-step clustering perfectly aligns with the “classification” objective (6). Secondly, this study includes both continuous variables (such as annual household income) and categorical variables (such as parental educational level) as indicators. The two-step clustering can simultaneously process both types of variables, thereby avoiding potential biases that might be introduced by preprocessing continuous variables through methods like normalization or standardization (7). Overall, this method can automatically and objectively categorize based on data characteristics, providing more intuitive and substantial evidence for understanding the diversity of family backgrounds and formulating differentiated intervention strategies.

### 1.1 The family economic environment

The family’s financial situation, parents’ level of education, and other factors make up the family economic environment (8). The relationship between the family economic environment and depression symptoms in adolescents has been thoroughly examined in a number of studies. Adolescents from families with high levels of family economic environment were two to three times less likely to experience mental health issues than those from families with low levels of family economic environment, according to the findings of a systematic review (9). Additionally, Katie A. et al. (10) demonstrated that adolescents who live in poor family economic environment—particularly those whose parents have low educational attainment—are more prone to experience depression. Whereas, other studies have found no significant association between family economic environment and depressive symptoms in adolescents (11).

### 1.2 The family emotional environment

The family emotional environment refers to the non-physical factors in the family that influence the emotional, psychological and behavioral development of family members (12). It is a dual-factor influence model, similar to an umbrella structure, encompassing a wide range of elements, such as the explicit environment (parental role modeling, family regulations) and implicit environment (family culture, parent-child relationship) (13). The family emotional environment, as the social environment within the family, is considered to be an essential factor in adolescent mental health, with intimacy and conflict playing the largest role in adolescent emotional problems (14). Adolescent depression may be triggered by inadequate family intimacy or emotional expression, according to Jiali Shi et al. (15), while Xian Li et al. (16) also proposed that adolescents’ internalizing behavioral issues may be positively impacted by high family functioning.

### 1.3 Other influences and depressive symptoms

During the middle school stage, adolescents experience a vigorous development of self-awareness, gradually detaching from their families and integrating into schools. Peer relationships are the most anticipated interpersonal relationships for teenagers (17). As an important part of adolescents’ social development, peer relationships provide emotional support and social opportunities, which help in forming a healthy self-identity and the ability to cope with stress. Additionally, with the development and increasing importance of the internet, its side effects have also raised growing public concern, such as internet addiction (18). Internet addiction is a way for teenagers to escape real-life pressures; excessive reliance on the internet can not only affect academic performance and physical health but may also lead to the deterioration of social skills and psychological issues. Therefore, paying attention to the peer relationships and internet usage behaviors of adolescents is crucial for promoting their mental health and social adaptability.

Previous studies have shown that depressive symptoms in adolescents are associated with peer relationships, internet addiction, and family environmental factors (19). Individual traits of adolescents are significant predictors of their problematic internet use, and the development of these traits is closely related to the family environment. Research indicates that parenting styles can shape the personality of adolescents and subsequently influence their internet addiction behavior. This provides a more nuanced mechanistic explanation for understanding the impact of the family emotional environment on the mental health of adolescents (20, 21). Internet addiction refers to pathological internet-dependent behavior without substance addiction (22). Based on a large-scale cross-sectional study of 15,623 Chinese adolescents, 33.15% met the diagnostic criteria for internet addiction (23). It was discovered that adolescents with internet addiction frequently experienced anxiety and depressed symptoms, and that teenagers with a poor family atmosphere are more likely to be addicted to the internet (24).

Moreover, Susan G and other scholars discovered that parenting styles in the family environment have a significant impact on adolescents’ development, behavior, etc. Children raised with positive parenting practices are more likely to fit in with their peers (25). The quality of peer relationships is a significant predictor of the severity of depressive symptoms in adolescents, and negative peer relationships can significantly increase the risk of depression in adolescents (26).

Although studies have confirmed the correlation between family environment, internet addiction, and peer relationships with adolescent depressive symptoms, the concept of family environment is broad. Currently, few studies have separated the material and cultural aspects to explore which type of environment has a greater impact on adolescent depressive symptoms, and even fewer have comprehensively examined how these factors work together in adolescent mental health.

### 1.4 The current study

**Figure 1:**
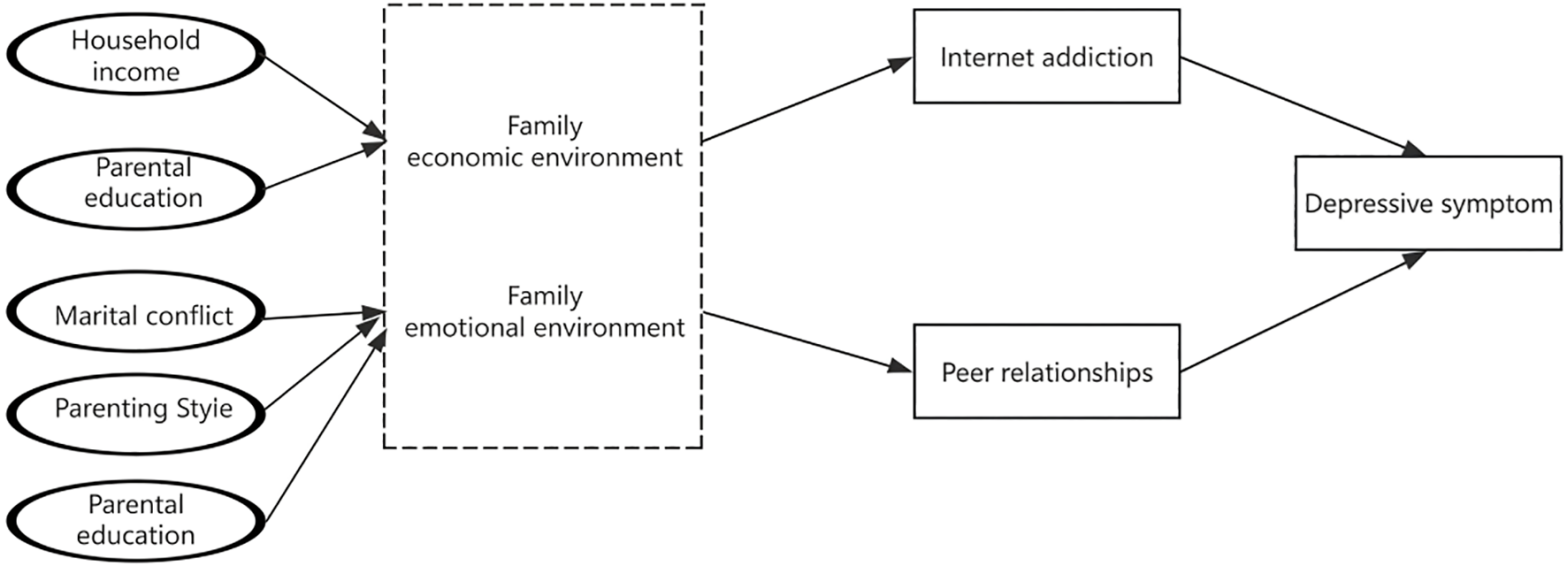
Proposed intermediary model.

To better understand the relationship between different family environments and adolescent depressive symptoms, this study aims to achieve three objectives. First, we investigate how to classify the family emotional environment and family economic environment and analyze the distribution of depressive symptoms across different family environments. This helps clarify the differential impacts of family emotional and economic environment on depressive symptoms, providing a scientific basis for developing precise mental health intervention strategies. Next, our goal is to examine whether different family environments are associated with depressive symptoms. Finally, this study will explore the pathways and conditions through which family environment influences adolescent depressive symptoms, using a mediation model (**Figure 1**) where internet addiction and peer relationships act as mediators.

### 1.5 The hypothesis of present study

This study is based on the Family System Theory (27), Social Ecology Theory (28), and Social Support Buffer Hypothesis (29), and proposes the following hypotheses:

Hypothesis 1a. The family emotional environment is negatively correlated with adolescent depressive symptoms.
Hypothesis 1b. The family economic environment is negatively correlated with adolescent depressive symptoms.
Hypothesis 2a. The influence of family emotional environment on adolescent depressive symptoms is mediated by internet addiction and peer relationships.
Hypothesis 2b. The influence of family economic environment on adolescent depressive symptoms is mediated by internet addiction and peer relationships.

## 2 Method

### 2.1 Participants

We randomly selected first-year students from four junior high schools in Nantong City and Yancheng City, Jiangsu Province, and randomly selected classes from this grade. Every student in the chosen classes was invited to take part in the study, and the schools organized and distributed the questionnaires. In the end, 2,825 students (94.17%) completed the questionnaires out of a total of 3,000 that were distributed. There were 1354 girls and 1471 boys among them.

The study was conducted in accordance with the Declaration of Helsinki as revised in 1989 and was approved by the Ethics Committee of Nantong University, the approval number is NO.(2024)04. Prior consent was obtained from all the participants and their guardians before the questionnaire work was carried out. The nature and purpose of the study were explained to them in detail and they were solemnly promised that all information collected would be kept strictly confidential. Each participant voluntarily spent 30 minutes in their classroom filling out the questionnaire. Questionnaires were not distributed when students had cognitive impairments or had been diagnosed with depression. At the end of the survey, students were presented with a small gift to show their appreciation and provide psychological feedback to the school as a class.

### 2.2 Measures

#### 2.2.1 Depressive symptom scale

The Children’s Depression Inventory (CDI) is a self-report measure used to assess depressed mood or behavior in the past two weeks in children and adolescents 7–17 years of age, developed by Kovacs (30) and consisting of 27 questions. On a scale of 0 to 2, each question is given a score of “occasionally,” “often,” and “always,” for a total of 54. A cut-off score of 19 was used to identify depressive symptoms. In this study, the Cronbach’s α of the scale was 0.92.

#### 2.2.2 The family emotional environment

The analytical dimensions of the family emotional environment mainly refer to Yufeng Guo’s methodology (31). The family emotional environment factors were evaluated using the Parenting Behavior Inventory (PBI), the Children’s Perceived Marital Conflict Scale(CPIC), the Father’s education, and the Mother’s education.

1. The Parenting Behavior Inventory (PBI) consists of the Father Parenting Behavior Inventory (PBI-F) and the Mother Parenting Behavior Inventory (PBI-M), which were developed by Parker in 1979 (32) to assess individuals’ perceptions of parenting behavior. The scale consists of 23 questions, each of which is rated on a scale of 0-3, indicating “very inconsistent”, “somewhat inconsistent”, “somewhat consistent” and “Very Compliant”, respectively. Better parenting practices are indicated with higher scores. In this study, the PBI-F scale’s Cronbach α was 0.71, while the PBI-M scale’s Cronbach α was 0.69.
2. The degree of perceived parental conflict among middle school adolescents was assessed using the Conflict Characteristics sub scale of the Children’s Perception of Interparental Conflict Scale (CPIC), which was updated by Liping Chi (33). The scale consists of 17 questions, each of which is rated from 1 to 4, indicating “completely consistent”, “relatively consistent”, and “relatively inconsistent”, respectively. The higher the total score, the higher the perceived level of parental conflict. The scale used in this investigation had a Cronbach α of 0.93.
3. Parental education was examined using a self-created questionnaire. Parental education was divided into four levels: elementary school and below, middle school, high school and junior college, and university (college) and above, which were scored as 1–4 respectively.

#### 2.2.3 The family economic environment

The analytical dimensions of the family economic environment draw reference from Bradley et al.’s methodology (34). The family economic environment factors such as the average annual household income and the parents’ education level, were examined using a self-designed questionnaire. With scores ranging from 1 to 4, the parental level of education was separated into four categories: elementary school and below, junior high school, high school and middle school, and university (college) and above. The average annual household income is categorized into three tiers, each worth 1–3 points: poor ($10,000–$80,000), middle ($80,000–$150,000), and good ($150,000–$300,000).

#### 2.2.4 Internet addiction diagnostic questionnaire

The Internet Addiction Diagnostic Questionnaire (YDQ) was developed by Kimberly S. Young (35) and consists of 8 questions, with a score of 1 for “yes” and 0 for “no”. The higher the total score, the higher the level of Internet addiction. In this study, the Cronbach’s α for the YDQ scale was 0.78.

#### 2.2.5 Peer relationships

The Peer Relationship Scale for Children and Adolescents (36) developed by Prof. Guo Boliang was used to respond to adolescents’ subjective experiences and feelings when interacting with others. Here are 22 questions in the survey, and there are four possible scores: 1= “Not like this”, 2= “Sometimes like this”, 3= “Often like this”, 4= “Always like this”. The individuals’ peer relationships status deteriorates with increasing overall score. The scale’s Cronbach α in this investigation was 0.90.

#### 2.2.6 Demographic variables

Demographic variables included gender, home location, and whether or not they were born alone.

#### 2.2.7 Statistical analysis

SPSS 26.0 and AMOS 26.0 were used to statistically analyze the data. All tests were two-tailed and the level of significance was set at *P* < 0.05. Before the main analysis, the variables were tested for common method bias using Harman’s one-way analysis of variance. The second-step clustering algorithm in SPSS was used to calculate the family emotional and economic environment’s factors. To ensure that all continuous variables are comparable in cluster analysis and to prevent bias caused by different scales, before clustering, we automatically performed Z-score standardization on the continuous variables using SPSS software, transforming them into a distribution with a mean Z of 0 and a standard deviation of 1. For categorical variables, the algorithm automatically processed them based on their inherent attributes. When determining the optimal number of clusters, the algorithm systematically compared a series of models ranging from 1 to the maximum number of clusters (N = 15) and calculated the fit indices for each model. We primarily used the Bayesian Information Criterion (BIC) to judge the change, selecting the model with the smallest BIC value or the model where the BIC change rate showed a significant drop as the optimal solution. At the same time, we referred to the silhouette coefficient to assess the overall quality of cluster cohesion and separation, where a silhouette coefficient close to +1 indicates better clustering results, and close to -1 indicates poorer clustering results. Pearson correlation analysis was utilized to test for correlations between variables. The AMOS model was used to test the mediating effect. The goodness-of-fit of the structural equation model was evaluated using a comprehensive set of indices, including the CMIN/DF, Comparative Fit Index (CFI), Root Mean Square Error of Approximation (RMSEA), and Standardized Root Mean Square Residual (SRMR). A model is generally considered to have a good fit when CMIN/DF < 5, CFI > 0.90, RMSEA < 0.08, and SRMR < 0.08. The significance of the effects was assessed using the bootstrap method (5000 replicated samples), and a 95% *CI* that did not contain 0 meant that the effect was significant.

## 3 Results

### 3.1 Common method bias test

All questionnaire items were subjected to exploratory factor analysis using Harman’s one-way method with unrotated principal component analysis. The results indicated that 18 factors in total had eigenroots greater than 1, and that the first factor explained 25.45% of the variance, which was less than the critical indicator of 40%. As a result, this study does not have a significant common method bias issue.

### 3.2 Sample characteristics

As shown in **Table 1**, this study included a total of 2,825 adolescents, including 1,471 males and 1,354 females. Among them, 1,328 were from urban areas and 1,597 were from rural areas. In terms of being an only child, 1,075 (38.05%) were only children, while 1,750 (61.95%) were not only children. Regarding family structure, 240 (8.50%) came from single-parent families, while 2,585 (91.50%) came from non-single-parent families.

**Table 1:** Demographic characteristics of the sample.

Variable	Category	N(%)
Gender	Male	1471(52.07%)
	Female	1354(47.93%)
Home location	City	1328(47.01%)
	Rural	1497(52.99%)
Is it an only child	Yes	1075(38.05%)
	No	1750(61.95%)
Family structure	Single parent	240(8.50%)
	Non-single parent	2585(91.50%)

### 3.3 The family emotional environment

**Figure 2:**
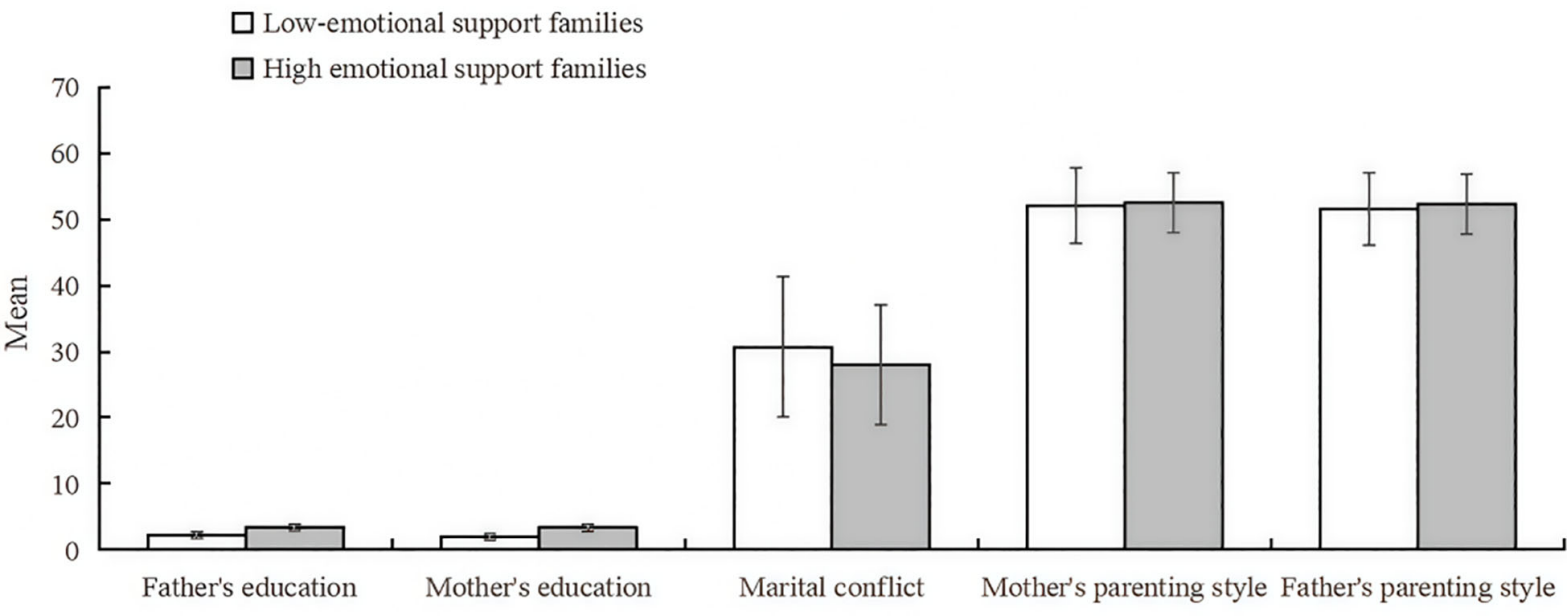
Second-step clustering results of family emotional environment factors.

**Figure 3:**
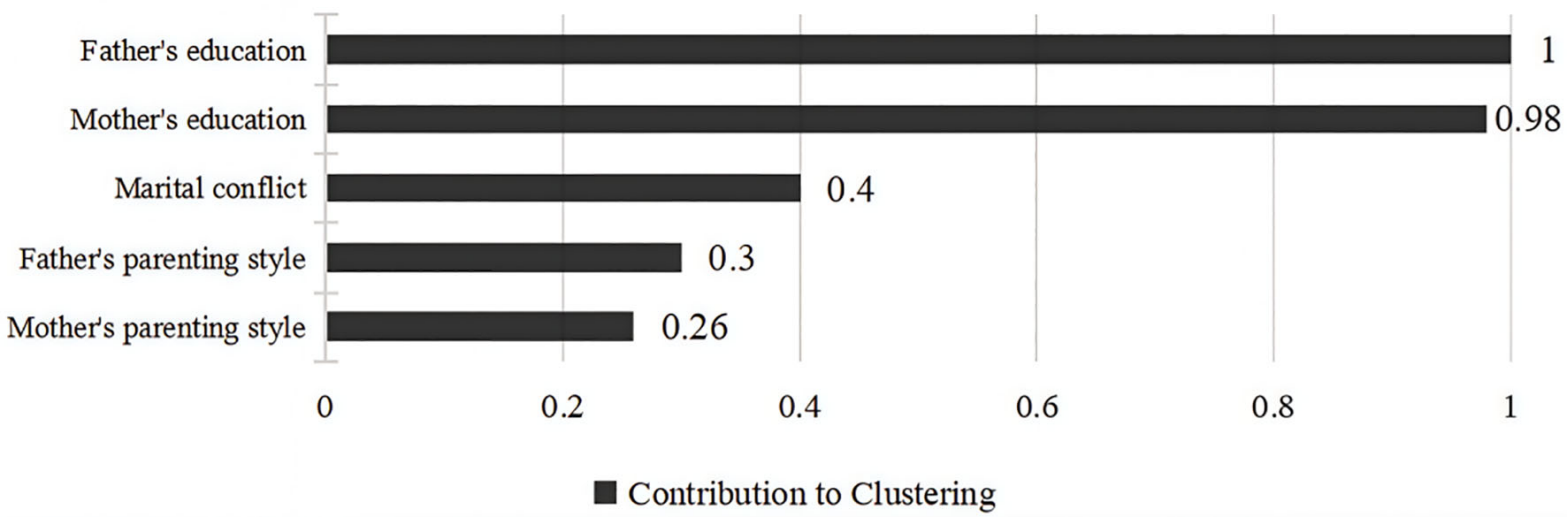
Degree of contribution of each variable to clustering.

The clustering profile measure was 0.5, indicating that the quality of this clustering was fair. Based on the BIC criterion, the optimal number of clusters for the family emotional environment was determined to be 2. The first category is low emotional support families, totaling 1481 (52.4%), with more detached family relationship, and the 2nd category is high emotional support families, totaling 1344 (47.6%), with more harmonious family relationship, and the specific results are shown in **Figure 2**. The degree of contribution of the 5 variables to the clustering is shown in **Figure 3**, in which “ father’s education level (V1) “ has the highest degree of contribution, and “ mother’s parenting style (V5) “ has the lowest degree of contribution.

### 3.4 The family economic environment

**Figure 4:**
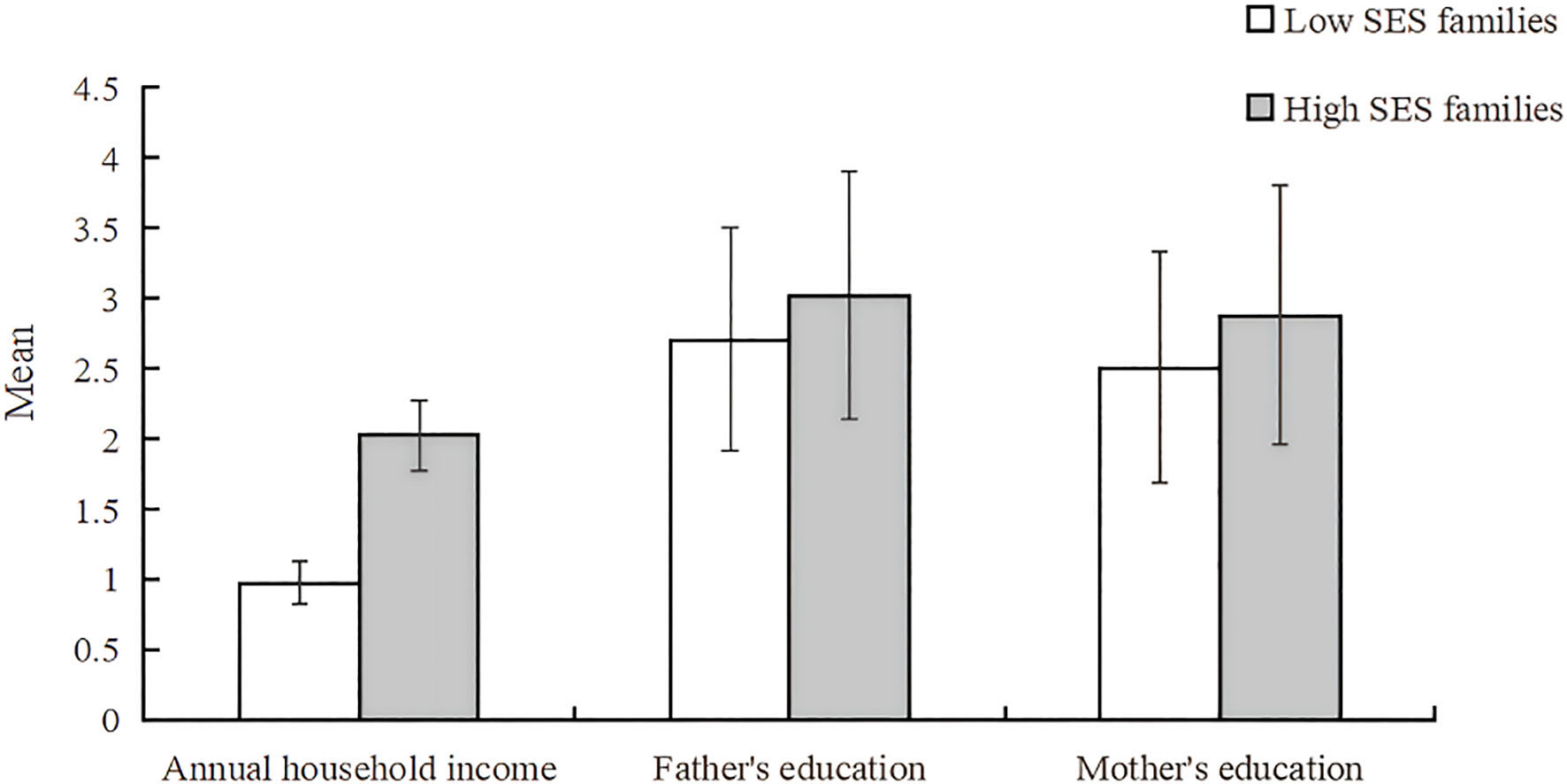
Second-step clustering results of the family economic environment factors.

**Figure 5:**
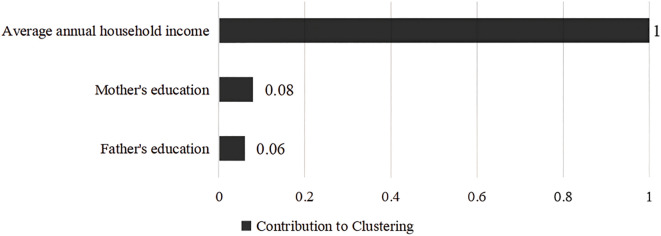
Degree of contribution of each variable to clustering.

The clustering profile measure was 0.4, indicating that the quality of this clustering was fair. According to the BIC quasi-test, the optimal number of clusters for the family economic environment was 2 categories, the first category is high family socioeconomic status (SES), a total of 800 (28.7%), family resources are more abundant, and the second category is low family socioeconomic status, a total of 1,985 (71.3%), the family resources are more scarce, see **Figure 4**. The contribution of the three variables to the clustering is shown in **Figure 5**, with “average annual household income” contributing the most and “father’s education” contributing the least.

### 3.5 Descriptive statistics between variables and correlation analysis

**Figure 6:**
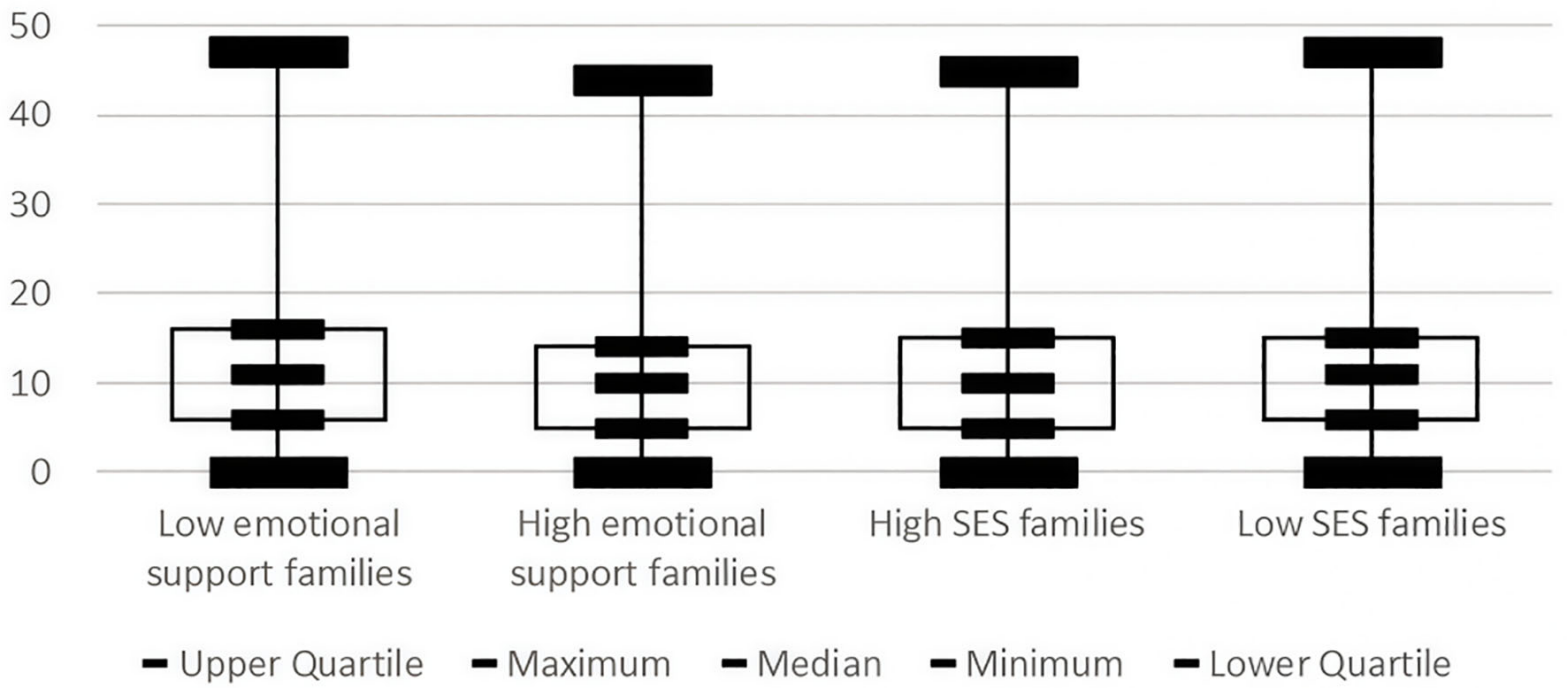
Distribution patterns of depressive symptoms in family environments.

**Figure 6** displays the depressive symptom distribution patterns in family emotional and economic environments. As shown, the distribution of depressive symptoms was more concentrated and had a lower median in families with higher emotional support, while the distribution of depressive symptoms was more dispersed and had a higher median for families with low emotional support, suggesting that emotional support may have a significant effect on the severity and distribution of depressive symptoms. The distribution of depressive symptoms was more concentrated and had a lower median in high SES families, while the distribution of depressive symptoms was more dispersed and had a higher median in low SES families, suggesting that SES may have a significant effect on the severity and distribution of depressive symptoms.

**Table 2** shows the correlation matrix of study variables, indicating strong correlations between peer relationships, internet addiction, depressive symptoms, and family emotional environment, with no associations found between these variables and family economic environment.

**Table 2:** Results of correlation analysis of research variables.

Variable	1	2	3	4	5	6	7	8	9	10
1 Depressive symptom	1									
2 Family emotional environment	-0.07^**^	1								
3 Family economic environment	0.01	-0.18^**^	1							
4 Father’s education	-0.01	0.73^**^	-0.17^**^	1						
5 Mother’s education	-0.04	0.78^**^	-0.19^**^	0.61^**^	1					
6 Marital conflict	0.50^**^	-0.13^**^	0.04	-0.06^**^	-0.07^**^	1				
7 Father’s parenting style	-0.08^**^	0.07^**^	-0.04	0.02	0.05^*^	-0.11^**^	1			
8 Mothers’ parenting styles	-0.09^**^	0.05^**^	-0.04^*^	-0.01	0.04^*^	-0.13^**^	0.81^**^	1		
9 Internet addiction	0.55^**^	-0.05^**^	-0.02	-0.01	-0.03	0.39^**^	-0.03	-0.02	1	
10 Peer relationships	0.69^**^	-0.05^**^	0.01	0.01	-0.01	0.49^**^	-0.08^**^	-0.09^**^	0.48^**^	1

^**^ indicates *P* < 0.01; ^*^ indicates *P* < 0.05. The bolded text indicates the correlation between key variables in this study.

### 3.6 Mediation analyses

In order to clarify the mechanism of different family environments on adolescents’ depressive symptoms, the present study further explored the mediating effects of internet addiction and peer relationships between the two based on correlation analysis. The correlation analysis showed that family economic environment was not related to depressive symptoms, which means it could not be mediated. Therefore, in this study, the family emotional environment was used as independent variable, internet addiction and peer relationships as mediating variables, depressive symptoms as dependent variables, and gender, home location, and whether or not they were born alone as control variables, and a multiple mediation model was constructed by using AMOS26.0.

Each parameter of the structural equation model was estimated in this study using the maximum likelihood technique. AMOS 26.0 processing produced the model’s final fit. As shown in **Table 3**, the model fit indices CMIN/DF=2.220, RMSEA = 0.021, CFI = 0.998, SRMR = 0.041, AGFI = 0.994, NFI = 0.996, and all the fit indices are within the reference range, which indicates that the model is well fitted.

**Table 3:** Model fit test (N = 2825).

Indicator	CMIN/DF	RMSEA	CFI	SRMR	AGFI	NFI
Reference point	1-3	<0.08	>0.95	<0.08	>0.9	>0.9
Actual value	2.220	0.021	0.998	0.041	0.994	0.996

**Figure 7:**
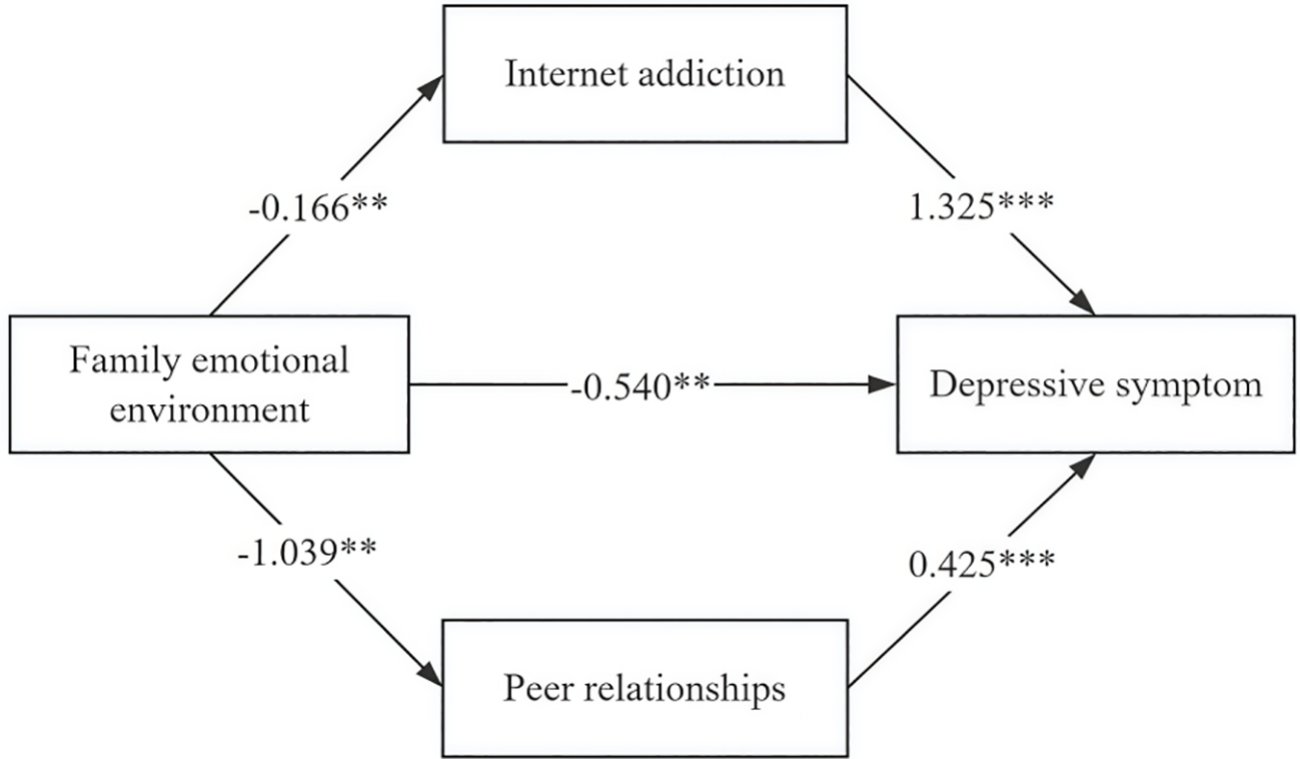
Parallel mediating model of family emotional environment, depressive symptoms, internet addiction, and peer relationships. (*** indicates *P* < 0.001, ** indicates *P* < 0.01).

After model fitting, the bias-corrected Bootstrap method (5,000 samples) confirmed significant parallel mediating effects of internet addiction and peer relationships. The results showed that the 95% confidence interval of the total indirect effect from family emotional environment to depressive symptoms was (-0.068, -0.015), and the interval did not contain 0, indicating that the parallel mediating effects of internet addiction and peer relationships were significant. Meanwhile, the 95% confidence intervals of the two pathways, family emotional environment — internet addiction— depressive symptoms and family emotional environment — peer relationships — depressive symptoms, were (-0.396, -0.060) and (-0.761, -0.117), respectively, and the intervals did not contain 0, suggesting that the mediating effect of internet addiction, peer relationships between the family emotional environment and depressive symptoms was also significant. The total effect of the family emotional environment on depressive symptoms was -1.20, with a direct effect of -0.54 (44.96%) and indirect effects via internet addiction (-0.22, 18.32%) and peer relationships (-0.44, 36.72%). The study partially confirmed that family emotional environment directly and negatively affect adolescents’ depressive symptoms, with indirect effects via internet addiction and peer relationships (**Table 4**, **Figure 7**).

**Table 4:** Parallel mediating effects of Internet addiction and peer relationships between family emotional environment and depressive symptoms.

Effect	Path	Estimate	Percentage of total effect (%)	Boot 95%*CI*	
				*LCI UCI*	
Direct effect	Family emotional environment→Depressive symptom	-0.54	44.96%	-0.060	-0.009
Indirect effect 1	Family emotional environment→ Internet addiction →Depressive symptom	-0.22	18.32%	-0.396	-0.060
Indirect effect 2	Family emotional environment → Peer relationships → Depressive symptom	-0.44	36.72%	-0.761	-0.117
Total indirect effect		-0.66	55.04%	-0.068	-0.015
Total effect		-1.20	100%	-0.109	-0.039

## 4 Discussion

### 4.1 The relationship between family environment and depressive symptoms

In the present study, the family emotional environment negatively correlated with adolescent depressive symptoms, supporting H1a. This finding aligns with previous research and is well explained by the Family systems theory, which posits that a positive family emotional environment enhances psychological resilience, whereas a negative one increases the risk of depression (37). In dysfunctional families, parental conflict, controlling parenting styles, and poor communication exacerbate adolescents’ negative emotions, leading to depressive symptoms (38). Excessive parental control can diminish adolescents’ autonomy and increases feelings of helplessness, while chronic criticism can damage children’s self-esteem and trigger negative emotions. Furthermore, parents with higher levels of education tend to communicate more effectively, offering emotional support and fostering self-confidence. Positive parent-child interactions encourage healthy attribution patterns (e.g., crediting success to effort), reducing depression and improving mental health (39).

In line with some earlier research, our data showed no significant correlation between the depressive symptoms of adolescents and family economic environment. A cross-sectional survey in China showed that a growth mindset can mitigate depressive symptoms in teens from low-SES families by fostering resilience (40). According to the Social Ecological Theory (41), the family is a microsystem that directly influences adolescents, while the family economic environment is an external system that indirectly influences. Children’s personalities and behavioral patterns are directly influenced by their parents, and children raised with firm yet lovingly supportive parenting typically grow up to be self-assured and socially adept. Therefore, compared with the family economic environment, the family emotional environment has a greater impact on adolescent depression. This study did not find a significant relationship between the family’s economic environment and adolescent depressive symptoms, a finding that diverges from both domestic and international research emphasizing the central role of socioeconomic status (42, 43). Firstly, in terms of measurement, there are differences in the tools used by various studies to assess depressive symptoms and the socioeconomic environment, which may affect the comparability of results. Secondly, at the sociocultural level, the sample of this study comes from Jiangsu Province, China, where the average household income and educational level are relatively high compared to the rest of the country. This relative homogeneity in the economic environment may have weakened the impact of family economic factors on the psychological health of adolescents. Additionally, due to cultural differences between the East and West, Chinese parents may often adopt traditional high control, low emotional response parenting styles, often ignoring or avoiding emotional issues that arise with their children, which differs from the low control parenting style of Western parents, leading to differences in responses to children’s psychological issues (44). At the same time, influenced by Confucian thought, Chinese parents tend to be more tolerant and restrained in marital conflicts, rather than actively resolving them, which contrasts with Western parents who are more likely to choose open communication to resolve conflicts (45). We acknowledge SES as an important variable that affects adolescent depressive symptoms, but due to the limitations of this study’s sample size and methodology, we were unable to determine whether there is a correlation between the family economic environment and adolescent depression. However, this study shows that the impact of the family emotional environment on depressive symptoms in Chinese adolescents is more significant.

### 4.2 The mediating role of internet addiction and peer relationships

Our research validated Hypothesis H2a, demonstrating that peer relationships and internet addiction mediate the link between adolescent depression symptoms and the family emotional environment. Prior studies reveal the family emotional environment’s crucial role in adolescent Internet use (46). Per the compensatory internet use theory (47), adolescents lacking parental warmth or care may seek affection online, heightening the risk of internet addiction. Moreover, a conflict-ridden family environment drives teens to escape into the virtual world. Excessive internet use weakens real-life social connections, impairs social skills, and exacerbates psychological problems like depression. Additionally, internet-addicted teens often have irregular lifestyles, with unhealthy habits directly harming their physical and mental health, increasing the likelihood of depressive symptoms.

Based on the parental spillover theory (48), children’s peer relationships are directly or indirectly related to parent-child relationships. Parental conflicts, specifically impairs the emotional bond between parents and children, triggering negative thoughts and behaviors that affect peer interactions. Academic achievement is key to Chinese student’s peer acceptance (49), and family relationship quality, including parenting styles and parental conflicts, significantly impacts academic performance. Poor parenting and parent-child disputes lower academic achievement, reducing peer acceptance and harming peer relationships. Therefore, the quality of the family emotional environment, especially parenting styles and parent-child relationships, is decisive for adolescent peer relationships development. The quality-stress model posits that negative peer relationships are a major source of life stress. Such stress hinders the development of personal skills, causes negative self-evaluation, and leads to depression. Peer antagonism can directly cause depression and also make teenagers feel lonely, exacerbating the risk. Bullied teens, often shunned by peers and lacking social support, are more prone to loneliness and despair.

### 4.3 Protective measures

In summary, the family emotional environment can not only directly affect adolescents’ depressive symptoms, but also indirectly through internet addiction and peer relationships. The family emotional environment is the cornerstone of adolescent mental health. As the primary space for emotional growth and learning social skills, the absence of a family environment can make teens more vulnerable to depression. Thus, fostering a positive family emotional environment is vital for teen mental health and social well-being. Based on these findings, we propose comprehensive interventions: 1. Promote family emotional communication and understanding through activities and training. 2. Educate teens on proper internet use at home and school and provide professional help for those at risk of addiction. 3. Encourage healthy peer relationships and positive social engagement. 4. Establish and improve teen mental health services covering crisis response, therapy, and follow-up care.

### 4.4 Research implications and limitations

The study analyzes how family environment influences adolescent depressive symptoms by constructing family emotional and economic environment indices, addressing the limitation of prior single-factor research to better explain causes and inform targeted interventions. In addition, this study focuses on seventh-grade students, a group of adolescents with a high incidence of depression, providing an important opportunity for early intervention. At the same time, in terms of scale selection, we used a variety of standardized scales, especially in the area of family emotional environment, and comprehensively used a variety of international scales for evaluation to ensure the scientific and reliable nature of the research results. However, this study still has some limitations. First, the cross-sectional design cannot establish causal relationships between variables. Second, although the family emotional and economic environment factors selected in this study are typical and representative, they do not cover all potentially relevant factors. Future research should incorporate more variables for deeper analysis. Third, most of the data in this study were based on self-assessment, which may have introduced a source of error. Fourth, this study was conducted in Jiangsu, China, and thus caution needs to be taken regarding the generalizability of the findings.

## 5 Conclusions

The study finds a significant negative correlation between adolescent depressive symptoms and the family emotional environment and highlights that peer relationships and internet addiction mediate this association. In light of the paper’s findings, we ought to concentrate on cultivating a positive family emotional environment to support teenagers’ mental health and overall development.

## Data Availability

The data analyzed in this study is subject to the following licenses/restrictions: To protect the privacy of the participants, the data will not be publicly disclosed. If needed, it can be obtained from the first author or the corresponding author. Requests to access these datasets should be directed to fenghaiqing1105@163.com.
